# Development and Validation of a Reversed-phase HPLC Method for the Determination of Hydroxybenzene in a Cream Formulation

**DOI:** 10.4103/0250-474X.70475

**Published:** 2010

**Authors:** G. A. Shabir

**Affiliations:** Oxford Brookes University, School of Life Sciences, Headington Campus, Oxford, OX3 0BP, United Kingdom

**Keywords:** Hydroxybenzene, method validation, pharmaceutical cream formulation, reversed-phase HPLC

## Abstract

A rapid, sensitive and specific reversed-phase high performance liquid chromatographic method with diode-array detection has been developed and validated for the determination of hydroxybenzene (0.494%, w/w) in a commercially available cream pharmaceutical formulation. Isocratic chromatography was performed on a C18 column with methanol-water 60:40 (v/v) containing 0.1% phosphoric acid (v/v) as mobile phase at a flow rate of 1.0 ml/min. UV detection was at 254 nm. Linearity of the method was excellent (r^2^ = 0.9999). The relative standard deviation values for intra- and inter-day precision studies were < 1% and the recovery of hydroxybenzene was >99%. The limit of detection and quantitation for hydroxybenzene was found to be 13.5 η g/ml and 2 μg/ml, respectively. The method was also validated for specificity and robustness. The method was found to be robust and can be reliably used to determine the hydroxybenzene content of marketed formulations.

Hydroxybenzene is widely used in the preparation of drugs, cosmetics including sunscreens[1], hair dyes, and skin lightening preparations[2]. It is the starting material in the medicinal formulation of aspirin, herbicide, creams and synthetic resins. Hydroxybenzene belong to a family of compounds especially harmful for the environment, owing to their toxicity and carcinogenic effect[3]. This compound can be the source of serious health hazards if released into the environment through accidental spillage or poor disposal[4–6]. Recently, the determination of such phenolic compounds has been paid much attention, and verities of analytical methods have been reported. The hyphenation of gas chromatography (GC) and mass spectrometry (MS) has proven powerful for phenolic compounds assay[7,8], but the cost is generally high. For only GC based analysis, the poor polarity and insufficient sensitivity of hydroxybenzene are troublesome[9–12] so the chemical derivatization, such as silylation[13] and estrification[14] is necessary, which is time-consuming and costly. The objective of this study was therefore to develop a simple, sensitive, robust and precise reversed-phase high performance liquid chromatography (RP-HPLC) method for the analysis of hydroxybenzene in pharmaceutical cream formulation without derivatization process.

Today, RP-HPLC is the most popular analytical technique for separating complex mixtures in the chemical, pharmaceutical and biotechnological industry. RP-HPLC is the opposite of normal-phase chromatography, with a nonpolar stationary phase and a polar, largely aqueous mobile phase. The most common stationary phases used are octadecyldimethyl (C_18_) phases with silica as the solid support. Silica has a small pH range (3 to 8) where mixtures can be separated without degradation of the column performance. Above pH 8, silica supports dissolve and destroy the column. Below pH 3, the silicon-carbon bond is cleaved, and the column is destroyed. The separation is achieved by analytes having different interactions with the stationary phase. In RP-HPLC, solutes are separated using their hydrophobicity. A more hydrophobic solute will be retained on the column longer than a less hydrophobic one. Also, polar solutes will interact with the silica surface to cause peak tailing. The mobile phase is one of the two components involved in the separation process. Water is generally one of the components of a binary mixture in RP-HPLC. Water is considered to be the weak component of the mobile phase and does not interact with the hydrophobic stationary phase chains.

The most popular organic modifiers used in RP-HPLC are methanol and acetonitrile. The organic modifier is the strong solvent in the mobile phase. The higher the concentration of organic modifier, the less retention an analyte will have. There is no doubt that, also today LC-UV diode-array detection (DAD) plays an important role (detection and peak-purity) in many research and development studies, and for a wide variety of routine analyses. The RP-HPLC method reported in this study was validated in accordance with the International Conference on Harmonization (ICH) guideline[15] and best practice[16,17]. Specificity, linearity, precision (repeatability and intermediate precision), accuracy, robustness, limit of detection and limit of quantitation were evaluated.

## MATERIALS AND METHODS

Methanol (HPLC-grade), hydroxybenzene (pure > 99.5%), phosphoric acid (H_3_PO_4_) were obtained from Sigma-Aldrich (Gillingham, UK). Distilled water was de-ionised by using a Milli-Q system (Millipore, Bedford, MA). The Knauer HPLC system (Berlin, Germany) equipped with a model 1000 LC pump, model 3950 autosampler, model 2600 photodiode-array (PDA) detector and a vacuum degasser was used. The data were acquired via Knauer ClarityChrom Workstation data acquisition software. The mobile phase consisted of a mixture of methanol-water (60:40, v/v) containing 0.1% phosphoric acid (v/v). The flow rate was set to 1.0 ml/min. The injection volume was 20 μl and the detection wavelength was set at 254 nm. RP-HPLC analysis was performed isocratically at room temperature (24±1°) using a Spherisorb C_18_(250 x 4.6 mm, 5 mm) column (Waters, Elstree, UK).

### Standard and sample preparation:

An accurately weighed amount (100 mg) of hydroxybenzene, standard was placed in a 100 ml volumetric flask and dissolved in a methanol (stock). A 4 ml aliquot of stock solution was transferred to a second 100 ml volumetric flask and diluted to volume with mobile phase, yielding a final concentration of 40 μg/ml. An accurately weighed amount (1.0 g) of sample cream into a 100 ml volumetric flask and 20 ml methanol was added. The mixture was heated on a water bath to boiling point. Firmly stopper the flask and shake vigorously to disperse the melted sample. The solution was then cooled to room temperature. Fifty milliliter of mobile phase was added to the flask and again heated the mixture to boiling point. Firmly stopper the flask, shake the mixture vigorously to disperse the sample. Then sample was cooled to room temperature and diluted to 100 ml with mobile phase, yielding a final concentration of 10 mg/ml. The sample was filtered through a qualitative filter paper No. 1 (Whatman, Lawrence, USA) and injected into the HPLC system.

## RESULTS AND DISCUSSION

**Fig. 1:**
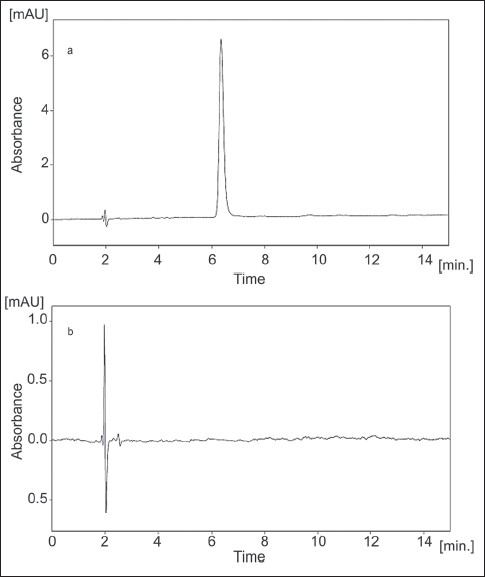
Typical LC chromatograms of sample and placebo Typical LC chromatograms obtained from, (a) sample; (b) chromatogram of placebo demonstrating the absence of interference with hydroxybenzene (retention time 6.3 min).

HPLC separation of hydroxybenzene was carried out on a Spherisorb C_18_ column by an isocratic elution with methanol-water (60:40, v/v) containing 0.1% phosphoric acid (v/v). The flow rate was constant at 1.0 ml/min and the column temperature was at room temperature (24±1°). The UV wavelength was set at 254 nm. No interference from diluents, impurities, or excipients present in the pharmaceutical formulation was observed at this detection wavelength. Before each run LC column was equilibrated with the mobile phase for about 15 min. A sharp, symmetrical peak was obtained for hydroxybenzene when analyzed under these conditions (fig. 1a). This retention time enable rapid determination of the drug, which is important for routine quality control analysis. Use of an un-buffered mobile phase is another advantage of the proposed method, because buffers reduce column life. Also sample preparation is simple and no dramatization process required.

System suitability test was established from six replicate injections of a solution containing 40 μg hydroxybenzene/ml. The percent relative standard deviation (RSD) of the peak area was calculated. The peak retention factor (k) for drug was calculated from k = (t_R_ – t_0_)/t_0_, where t_R_ and t_0_are the retention times of the peak of interest and the solvent front, respectively. A useful and practical measurement of peak shape, the peak asymmetry factor, A_s_, was calculated at 10% of peak height. Column plate number was determined using the formula, N = 5.54(t_R_/w_h_)^2^, where w_h_is the bandwidth at 50% of peak height. The proposed method met these requirements within the United States Pharmacopoeia (USP) accepted limits (Table 1)[18]. The stability of hydroxybenzene in solution was investigated in the method development phase. Six solutions containing 40 μg/ml of hydroxybenzene were tested. Analysis was performed after preparation and at 3 and 5 days thereafter. The solutions were stable during the investigated 5 days and the RSD was < 1.0% for retention time (min), peak area and height. Standard solutions stored in a capped volumetric flask on a laboratory bench under normal lighting conditions for 5 days, were shown to be stable with no significant change in hydroxybenzene concentration over this period.

**TABLE 1:** SEPARATION CHARACTERISTICS OF HYDROXYBENZENE ANALYSED UNDER OPTIMISED CONDITIONS

Parameter	Recommended limitsa^a^	Results
Retention time (min)	-	6.350
Injection repeatability^b^	RSD ≤ 2 % for n = 5	0.16
Retention factor (*k*)	K > 2.0	4.16
Width at 50% height (min)	-	0.18
Asymmetry factor (10%, A_s_)	A _s_≤ 2.0	1.10
Plate number (*N*)	N > 2000	11345

a United States Pharmacopeia limits

b Six replicate injections

Appropriate amounts of hydroxybenzene stock solutions (250 μg/ml) were diluted with mobile phase to give concentration of 2, 20, 30, 40, 50, 60 and 80 μg/ml. Each solution was injected in triplicate and calibration plot was prepared. Linearity was evaluated by linear least-squares regression analysis. Good linearity was observed over the concentration range evaluated (2-80 μg/ml). The slope and intercept of the calibration plot (±standard deviation, n= 3) were 158.45+1661.8 and the correlation coefficient was 0.9999 (Table 2). The validity of the assay was verified by analysis of variance. This revealed the regression equation was linear (F_calculated_ = 13,427 > F_critical_= 4.96; P = 5%) with no deviation from linearity (F_calculated_ = 0.14 < F_critical_ = 2.68; P = 5%).

**TABLE 2:** METHOD VALIDATION OF HYDROXYBENZENE

Validation step	Parameters	Results	Acceptance criteria
Linearity (*n* = 7)	Correlation coefficient (r^2^)	r^2^=0.9999	> 0.998
Repeatability (*n* = 6)	%RSD: t_R_ (min); Peak area	0.08 0.11	≤ 2
Intermediate precision (*n* = 3)	%RSD: Instruments; Analysts	0.16 0.12	≤ 2
LOD	s/n ratio	s/n = 3.06 (13.5 ηg/ml)	s/n ≈3:1
LOQ*	s/n ratio	s/n = 10.42 (2μg/ml)	s/n ≈10:1

* RSD (%); (n = 6); 0.24

The precision of the method was investigated with respect to repeatability and intermediate precision. The repeatability (intra-day precision) of the method was evaluated by assaying six replicate injections of the hydroxybenzene at 100% of test concentration (40 μg/ml) on the same day. The %RSD of the retention time (min) and peak area were calculated (Table 2). Intermediate precision (inter-day precision) was demonstrated by two analysts using two HPLC systems and evaluating the relative peak area percent data across the two LC systems at three different concentration levels (50%, 100%, and 150%) that cover the assay method range (2-80 μg/ml). The %RSD across the systems and analysts were calculated from the individual relative percent peak area mean values at the 50%, 100%, and 150% of the test concentration. The intra-day (*n*= 6) and inter-day (*n*= 3) %RSD are given in Table 2. All the data are within the acceptance criteria of 2%.

Accuracy of the method was evaluated by fortifying a hydroxybenzene sample solution (80 μg/ml) with three known concentrations of reference standard (30, 40, and 50 μg/ml). Percent recoveries were calculated form differences between the peak areas obtained for fortified and unfortified solutions. Good recoveries were obtained (Table 3). No significant differences were observed between amounts of hydroxybenzene added and the amounts found (*p* < 0.05).

**TABLE 3:** RECOVERY STUDIES OF HYDROXYBENZENE FROM SAMPLES WITH KNOWN CONCENTRATIONS

Validation step	% of nominal	Amount of analyte (μg/ml)		Recovery (%)*	RSD (%)*
		Added	Found		
1	50	4.0	3.98	99.50	0.07
2	100	8.0	7.98	99.75	0.10
3	150	12.0	12.01	100.08	0.09

* n = 3

Injections of the extracted placebo were performed to demonstrate the absence of interference with the elution of the hydroxybenzene. This result demonstrates (fig. 1b) that there was no interference from the other materials in the cream formulation and, therefore, confirms the specificity of the method. The forced degradation studies were also applied to hydroxybenzene reference standard at a concentration of 40 μg/ml to verify that none of the degradation products interfered with quantitation of the drug. Hydrolytic degradation was studied by heating the drug under reflux at 80° in 0.1 M hydrochloric acid and 0.1 M sodium hydroxide for 4 h. The samples were then cooled to room temperature and neutralized. Oxidative degradation was studied by treating the drug with 3% hydrogen peroxide at room temperature (24±1°) for 4 h. Solutions containing 40 μg/ml of each degraded sample were prepared and injected in triplicate. Thermal degradation was studied by heating samples in oven at 80° for seven days. No significant degradation was observed under any stress conditions studied (Table 4). In addition, DAD data were collected for all the degradation conditions studied, with the purpose of peak-purity evaluation. These analyses showed that no impurities or degradation products were coeluting with the hydroxybenzene peak.

**TABLE 4:** RESULTS OF THE STRESS CONDITIONS EXPERIMENTS

Stress conditions	Sample treatment	t_R_ (min)	Area (mAU.s)	Assay (%)*
Reference Standard	Fresh solution (40 μg/ml)	6.350	7928	99.97
Acid degradation	1M HCl at 80° for 4 h	6.348	7922	99.82
Base degradation	1M NaOH at 80° for 4 h	6.347	7917	99.71
Oxidative degradation	3% H_2_O_2_ at room temperature for 24 h	6.351	7925	99.85
Thermal degradation	Oven at 80° for 7 days	6.346	7921	99.81

* n = 6

**Fig. 2:**
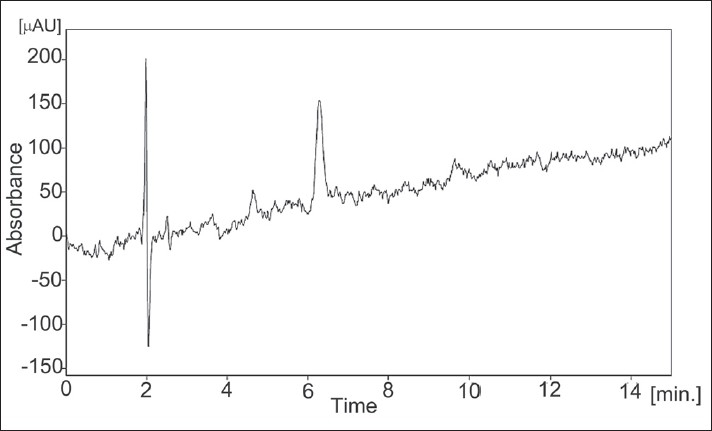
LC chromatogram for limit of detection for hydroxybenzene LC chromatogram for limit of detection for hydroxybenzene. Sample concentration 13.5 ng/ml.

The limit of detection (LOD) and limit of quantitation (LOQ) tests for the procedure were evaluated by serial dilutions of hydroxybenzene stock solutions in order to obtain signal-to-noise ratios (s/n) of ≈3:1 and ≈10:1, respectively. The LOD value for hydroxybenzene was found to be 13.5 ηg/ml (s/n = 3.06, fig. 2) and LOQ (*n* =6) was 2 μg/ml (s/n = 10.42) as shown in Table 2.

Robustness of the method was evaluated by the analysis of hydroxybenzene under different experimental conditions such as changes in the composition of the mobile phase, column temperature, flow rate and different columns. The percentage of methanol in the mobile phase was varied ±2%, the column temperature was varied ±3° and, the flow rate was varied ±0.2 ml/min. Their effects on the retention time (*t*_R_), asymmetry factor (*A*_s_) at 10%, recovery and repeatability were studied. Deliberate variation of the method conditions had no significant effect on assay data or on chromatographic performance, indicating the robustness of method and its suitability for routine use and transfer to other laboratories. The results from robustness testing are presented in Table 5.

**TABLE 5:** ROBUSTNESS OF THE METHOD

Conditions	Value	A_s_	t_R_ (min)	% (mean±sem)^a^	RSD (%)
Mobile phase (% methanol, ±2%)	58	1.11	6.352	99.9±0.1	0.10
	60	1.10	6.350	99.8±0.1	0.07
	62	1.10	6.348	99.8±0.2	0.11
Column temperature (±5°)	21	1.12	6.350	99.5±0.5	0.07
	24	1.10	6.350	99.7±0.3	0.05
	27	1.10	6.349	99.7±0.2	0.11
Flow rate (ml/min)	0.8	1.11	6.351	99.8±0.5	0.09
	1.0	1.10	6.350	100.0±0.2	0.11
	1.2	1.10	6.349	99.6±0.3	0.14
Lichrosorb C_8_ (250×4.6mm, 5 μm)		1.11	6.360	99.5±0.4	0.16

a Sem is the standard error of the mean, The recommended chromatographic conditions were Spherisorb C_18_ (250×4.6 mm, 5 mm) column with methanolwater 60:40 (v/v) mobile phase at a flow rate of 1 ml/min, UV detection at 254 nm and column temperature 24°

A RP-HPLC method with UV-DAD detection for the assay of hydroxybenzene was developed and validated. The results showed that the method is very selective, no significant interfering peak was detected; accurate, with the percentage recoveries >99; and reproducible, with the %RSD < 1%. The method was sensitive; a little as 13.5 ηg/ml could be detected with the LOQ of 2 μg/ml. The method involves use of a simple mobile phase and minimum sample preparation, encouraging its application in quality control for analysis of hydroxybenzene in bulk samples, raw materials and final cream products formulations.
